# Comparison of concept recognizers for building the Open Biomedical Annotator

**DOI:** 10.1186/1471-2105-10-S9-S14

**Published:** 2009-09-17

**Authors:** Nigam H Shah, Nipun Bhatia, Clement Jonquet, Daniel Rubin, Annie P Chiang, Mark A Musen

**Affiliations:** 1 Centre for Biomedical Informatics, Stanford University, Stanford, CA 94305, USA; 2 Department of Computer Science, Stanford University, Stanford, CA 94305, USA

## Abstract

The National Center for Biomedical Ontology (NCBO) is developing a system for automated, ontology-based access to online biomedical resources (Shah NH, *et al*.: **Ontology-driven indexing of public datasets for translational bioinformatics**. *BMC Bioinformatics *2009, **10(Suppl 2)**:S1). The system's indexing workflow processes the text metadata of diverse resources such as datasets from GEO and ArrayExpress to annotate and index them with concepts from appropriate ontologies. This indexing requires the use of a concept-recognition tool to identify ontology concepts in the resource's textual metadata. In this paper, we present a comparison of two concept recognizers – NLM's MetaMap and the University of Michigan's Mgrep. We utilize a number of data sources and dictionaries to evaluate the concept recognizers in terms of precision, recall, speed of execution, scalability and customizability. Our evaluations demonstrate that Mgrep has a clear edge over MetaMap for large-scale service oriented applications. Based on our analysis we also suggest areas of potential improvements for Mgrep. We have subsequently used Mgrep to build the Open Biomedical Annotator service. The Annotator service has access to a large dictionary of biomedical terms derived from the United Medical Language System (UMLS) and NCBO ontologies. The Annotator also leverages the hierarchical structure of the ontologies and their mappings to expand annotations. The Annotator service is available to the community as a REST Web service for creating ontology-based annotations of their data.

## Introduction and background

There continues to be a tremendous increase in the amount, diversity, and rate of generation of high-throughput datasets as well as exponential growth in the biomedical literature. Since 1999, Gene Ontology (GO) annotations of gene products have enabled queries to accurately identify gene products associated with a particular cellular component, biological process or a molecular function. Similarly, creation of annotations for public data resources based on other shared ontologies would enable researchers to locate datasets, tissue samples, and clinical trials that relate to a given disease. This capability would permit a whole new class of integrative analyses [1]. However, due to the size of the data and the complexity of the task involved, adding ontology-based annotations to online data repositories manually on a case-by-case basis is unlikely ever to scale [2].

At the National Center of Biomedical Ontology (NCBO), we are developing methods to annotate large numbers of data resources automatically, and have developed a prototype system for ontology-based annotation and indexing of biomedical data [3]. The key functionality of this system is to provide a service that enables users to locate biomedical data resources related to particular ontology concepts. The system processes the textual metadata of diverse biomedical data resources (such as gene-expression data sets, descriptions of radiology images, clinical-trial reports, and PubMed abstracts), annotating and indexing them with concepts from appropriate ontologies.

A critical step that our system performs is to recognize a given ontology concept in the text metadata of a record in some online data resource. This task is generally referred to as concept recognition. A core aspect of concept recognition is a lexicon (or dictionary) usually derived from taxonomy or ontology to which text is mapped. In the biomedical domain, the United Medical Language System (UMLS) is an extensive resource that incorporates a number of disparate terminologies and ontologies and that provides a cross-referencing of related concepts. However, efforts to map public, open biomedical resources to semantically rich thesauri such as the UMLS metathesaurus have been scattered. Barring a few initiatives, [1,4] most efforts to date have focused on mapping text from patient records to UMLS, rather than on mapping metadata from online biomedical resources [5,6].

Most previous work in concept recognition in bioinformatics has been restricted to the identification of protein and gene names [7-9], with a few groups attempting to identify concepts representing relationships among entities [10]. This trend is obvious when looking at popular tools such as EBIMed and TextPresso, all of which identify genes or proteins in documents, but struggle to identify disease names [10,11]. The same emphasis was visible in the BioCreative text-processing challenge, which was primarily concerned with recognizing gene and protein names [7].

In the field of clinical informatics, the efforts to recognize concepts in text have focused on finding disease names in electronic medical records, discharge summaries, clinical guideline descriptions, and clinical-trial summaries [5,6,12]. However, electronic medical records are seldom made "public" as online biomedical resources. As a result, current methods and tools are usually not portable across a different problem category – such as processing the metadata of public, open biomedical resources.

In recent times, there has been a shift in the focus of research from individual genes and proteins to entire biological systems [13]. As a result, researchers need services that can processes the metadata of diverse resources to annotate and index them with concepts from appropriate ontologies, and that can enable the researchers to locate resources related to particular ontology concepts. Concept recognition is a key step for such systems.

NLM's MetaMap was one of the first tools for recognizing UMLS concepts [14]. It is widely regarded as the gold standard for this task. Recently, there have been a number of tools such as Mgrep [15] and MTag [16] that also perform concept recognition. The advent of these new tools has made the task of evaluating concept recognizers particularly important.

We conducted a survey of existing concept recognizers based on their published reports, and selected MetaMap and Mgrep as the two tools to evaluate for our purposes. This paper provides comparison of NLM's MetaMap and the University of Michigan's Mgrep [15]. We choose Mgrep because it is claimed to be a fast and scalable tool for concept recognition with a high degree of customizability vis-à-vis dictionaries and resources. Considering the vast number of biomedical resources and ontologies available, factors of speed, scalability, and customizability are of prime concern in developing a concept-recognition system.

In the remaining part of the paper, we first give a brief outline of the concept-recognition task and discuss our data sources and dictionaries. We explain the evaluation methodology adopted and the results obtained. We then discuss the performance of concept recognizers based on a number of performance metrics such as precision and recall. We also analyze the suitability of a concept recognizer based on a number of subjective parameters such as ease of use, ability to customize, and scalability. We then describe how we used Mgrep to build the Open Biomedical Annotator Web Service. We conclude with a summary of our findings.

### Concept recognition

**Figure 1:**
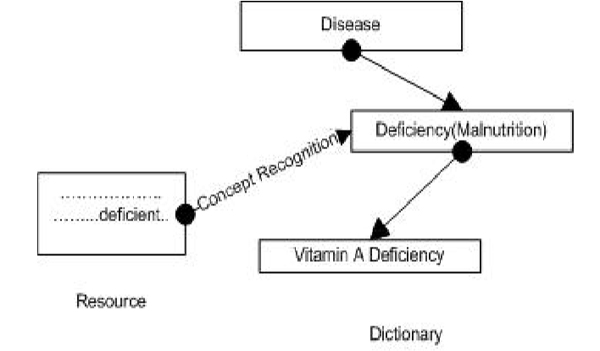
**Concept recognition**. The figure shows the working of a generic concept recognizer, which maps the text 'deficient' to the concept of 'Deficiency' in a hierarchical dictionary of concepts.

In the domain of biomedical informatics, the task of concept recognition can be understood as mapping biomedical text to a representation of biomedical knowledge consisting of inter-related concepts, usually codified as an ontology or a thesaurus. Figure 1 illustrates the task of a concept recognizer. Most concept recognizers take as input a resource and a dictionary – which can be a flat list or taxonomy of hierarchically related terms – and produce annotated files. The concept recognizer in Figure 1 recognizes the string 'deficient' in the resource and maps it to the concept 'Deficiency' in the dictionary. Most concept recognizers leverage natural-language processing and computational linguistic techniques to some extent.

## Methods

### Data sources

There are many online resources in biomedicine, ranging from data repositories such as Array Express and the Gene Expression Omnibus (GEO), radiology image repositories such as GoldMiner, which stores published images and their figure captions, to clinical trial repositories and Medline. Each of these resources assumes a particular type of biomedical knowledge. Comprehensive evaluations of concept recognizers would require several of these resources and their annotations to be evaluated. Also, it would be important to find out if a particular concept recognizer is more efficient in processing the textual annotations of certain resources. The variation in the sizes of the resources helps us to compare the scalability of a concept recognizer. For example, the size of the entire MedLine download is ~10.4 Gigabyte, while the size of ClinicalTrials.gov is only of the order of 99 Megabytes. This variation allows a performance benchmark on the scalability of the concept recognizers as well as an evaluation of the effect of data size on the execution time. Due to the generally large size of most biomedical resources, it is very important to see how scalable a concept recognizer is with respect to size. Table 1 gives a brief overview of the data resources we used in evaluating the concept recognizers. In each case, we used the title and description of an element from the resource as our input text for concept recognition.

**Table 1:** Size and number of elements of data sources

**Resource**	**Elements**	**Size**
ClinicalTrials.gov	50303	99 mb
Gold Miner (Subset)	2085	0.5 mb
Gene Expression Omnibus	2085	0.7 mb
PubMed (Subset)	2827	3.7 mb

### Dictionaries

A dictionary with respect to a concept recognizer is the set of terms or concepts that we aim to recognize in the data. Dictionaries from concept recognition are most commonly derived from taxonomies and ontologies about the domain of interest. Analogous to data sources, dictionaries can be specialized along different axes, such as diseases and anatomical parts. As most of the work in biomedical informatics has primarily focused in recognizing genes or proteins [7] the dictionaries for genomics and proteomics are comprehensive and extensively evaluated. The same is not true for dictionaries pertaining to diseases, body parts, biological processes, drug names, and so on.

We performed evaluations of concept recognizers using a number of different dictionaries. Thus, we could identify if a particular concept recognizer-dictionary combination is best suited for a particular semantic class of entities, such as diseases or body parts. Further, as in the case of data-sources, varying size of the dictionary helps to evaluate the scalability of the concept recognizers. As the data and the dictionary are both critical inputs to a concept recognizer, we note the effect of sizes of both in the performance of concept recognizers. In tune with the above notions – we performed evaluations using four different dictionaries (Table 2) of varying sizes. The 'diseases' dictionary comprises all the concepts in the UMLS that are of semantic type *disease or syndrome*. The 'biological processes' dictionary comprises all the GO biological processes contained in the UMLS.

**Table 2:** The size and number of concepts in each of the dictionaries SNOMED-CT, Diseases, FMA and Biological Processes (from GO)

**Dictionary**	**Size**	**Concepts**
SNOMED-CT	48 MB	1,139,586
Diseases	38 MB	764,420
FMA (Body Parts)	4.8 MB	93,335
Biological Processes	1.18 MB	31,294

### Evaluation workflow

We constructed a workflow for performing the evaluations and to provide a platform to plug in the data sources, on which to run the concept-recognition tools and to map the tool-specific output to a common format. Ideally, this task should have been done using a framework such as IBM's UIMA, but both MetaMap and Mgrep are not available as UIMA components. First, we randomly selected 200 lines from each data source and converted these sources from their native format to a format suitable for input to the concept-recognition tool. For example, the Array Express data are commonly available in XML format; however, University of Michigan's Mgrep requires the data to be in a three-column tab-delimited format. The next step involved running the concept-recognition tool and obtaining the processed file in a format specific to each tool. In the final step, we converted the output files of the different concept recognizers to a common format to ensure uniformity and to aid in performing comparative analysis. A total of four experts examined the resultant files for scoring true positives and false positives. We attempted to estimate recall by assuming a false negative result if no concept was identified. In addition, our evaluation considered customizability and scalability.

#### Customizability

We define the qualitative measure of customizability of a concept recognizer as the ease with which a dictionary and a data source can be configured for it.

#### Scalability

We define scalability by how easily a concept recognizer handles different sizes of dictionary and resource.

## Results

Tables 3 and 4 provide the numbers of concepts recognized by the two tools with different dictionaries and different data sources as input. Both tools recognize concepts from all resources tested and using all four dictionaries tested. In general, Mgrep recognizes a lesser number of unique concepts than MetaMap.

**Table 3:** Total number of concepts recognized by Mgrep and MetaMap across all resources using the biological process and diseases dictionaries

**Resource**	**Biological Process**		**Diseases**	
	
	**MG**	**MM**	**MG**	**MM**
Clinical Trials	10	106	409	710
Gold Miner	12	80	753	1283
GEO	136	188	337	704
MedLine subset	26	48	22	209

MG = Mgrep; MM = MetaMap

**Table 4:** Total number of concepts recognized by Mgrep and MetaMap across all resources using the Foundational Model of Anatomy and SNOMED-CT as dictionaries

	**FMA (Body Parts)**		**SNOMED**	
	
	**MG**	**MM**	**MG**	**MM**
Clinical Trials	243	380	1548	1730
Gold Miner	671	1097	3747	3400
GEO	272	818	2228	2372
MedLine subset	57	132	1320	1088

MG = Mgrep; MM = MetaMap

Table 5 compares the precision for the two tools using the Biological Processes dictionary from the Gene Ontology. To compute recall accurately requires the domain expert to go through each record to identify true and false negatives. We examined the option of estimating recall under the simplifying assumption that a concept should be recognized for every record processed and, if no true positive concept is recognized, then the record constitutes a false negative. This assumption could provide us with an estimate of the lower bound on recall [17]. However, this assumption does not hold for our current work; for example, for dictionaries such as Biological Processes and resources such as figure captions from radiology images, this assumption is flawed because there is no expectation that a biological process term will be mentioned in the figure caption of a radiology image. Therefore we were unable to estimate recall in a reliable manner.

**Table 5:** Precision of Mgrep and MetaMap using Biological Processes as the dictionary

**Data Source**	**Mgrep**	**MetaMap**
Clinical Trials	0.6	0.63
Gold Miner	0.58	0.33
GEO	0.93	0.73
MedLine	0.77	0.76

Table 6 compares the precision for the two tools using the 'diseases' dictionary, which contains UMLS concepts that are of semantic type *disease or syndrome*. We are unable to calculate recall in this case as well because some concept was recognized in almost all records and we cannot estimate recall using the assumption discussed above.

**Table 6:** Precision of Mgrep and MetaMap using the 'diseases' dictionary

**Data Source**	**Mgrep**	**MetaMap**
Clincal Trials	0.87	0.71
Gold Miner	0.73	0.548
GEO	0.88	0.755
MedLine	0.23	0.091

In general, Mgrep has a higher precision in recognizing Biological Processes. When considering precision, Mgrep outperforms MetaMap in almost all cases, with the exception for MetaMap in recognizing Biological Processes in records from ClinicalTrials.gov.

## Building the Open Biomedical Annotator

Currently, there are over 1000 public biomedical data resources listed in the *Nucleic Acids Research *(NAR) online Molecular Biology Database Collection. There are many more that are not listed by NAR. Across all such databases, ontology based annotation of their records is not as widespread as desired. There are several reasons for this limitation:

• Annotation often needs to be done manually either by expert curators or by the authors of the data (e.g., when a new Medline entry is created, it is manually indexed with MeSH terms);

• The number of biomedical ontologies available for use is large; ontologies change often and frequently overlap. The ontologies are not in the same format and are not always accessible via Application Programming Interfaces (APIs).

• Annotation is often a boring additional task without immediate reward for the user.

Even though ontologies are available as a one-stop-shop via BioPortal [18], the task of actually using the ontologies for annotation is non-trivial. Moreover, it has been shown that manual annotation efforts are unlikely to scale and that automated methods are required [2]. One of the core aims of NCBO is to provide annotation tools that enable the use of ontologies for annotation and that reduce the manual overhead for creating ontology-based annotations.

Based on the results of our comparison between Mgrep and MetaMap (see discussion), and because Mgrep had significantly faster execution time and can work with non-UMLS dictionary sources (which MetaMap cannot), we decided to use Mgrep as the initial concept recognizer for building the Open Biomedical Annotator Web service. A detailed description of the Annotator Web service is provided in [19]; we briefly review the key features here.

The Annotator service: (1) processes the raw textual metadata of online biomedical resources and tags them with relevant biomedical ontology concepts and (2) returns the annotations to end users. The Annotator Web service allows end users to utilize ontologies for annotation of biomedical data with minimal effort.

**Figure 2:**
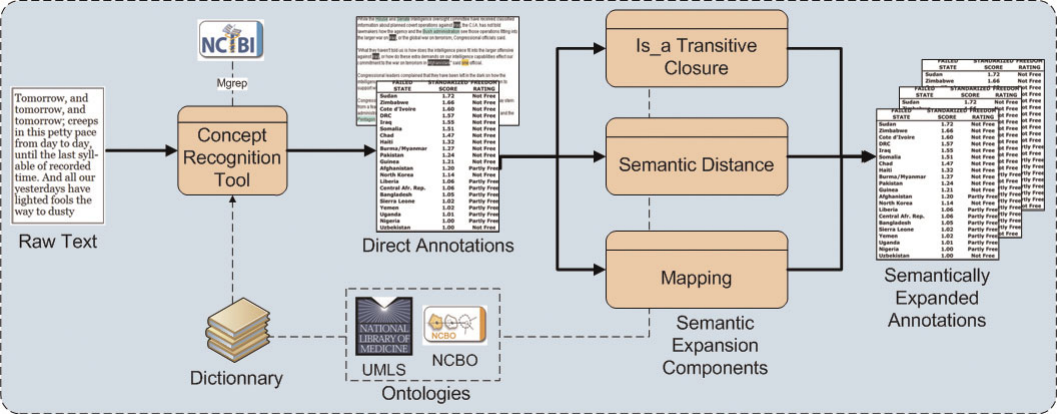
**Annotator Web service workflow**. The figure shows the Annotator Web service workflow. First, direct annotations are created from raw text based on syntactic concept recognition according to a dictionary that use terms (concept names and synonyms) from both UMLS and NCBO ontologies. Second, different components expand the first set of annotations using ontology semantics (e.g., subsumption relationships and mappings between ontologies).

The Annotator Web service's workflow is composed of two main steps (Figure 2). First, the user's free text is given as input to a concept recognition tool – Mgrep, developed by the University of Michigan's National Center for Integrative Biomedical Informatics – along with a dictionary. The dictionary (or lexicon) is a list of strings that identifies ontology concepts. The dictionary is constructed by accessing biomedical ontologies and pooling all concept names or other string forms, such as synonyms or labels that syntactically identify concepts.

The choice of the set of ontologies used to create the dictionary depends of the type of biomedical data the Web service is used to annotate. For instance, if a user wants to annotate gene-expression datasets with disease names, then SNOMED-CT and the NCI Thesaurus could be used. The output of the first step is a set of direct annotations.

This primary set of annotations serves as input for the semantic expansion components, which enhance the annotations extracted from the first step using the hierarchical structure of ontologies as well as mappings between them. For example: an is-a transitive closure component traverses an ontology parent-child hierarchy to create new annotations with parent concepts. For instance, if data are annotated with a concept from the NCI Thesaurus, such as *melanoma*, this component generates a new annotation with the term *skin neoplasm*, because the NCI Thesaurus provides the knowledge that melanoma is a kind of skin neoplasm. A semantic-distance component uses a given notion of concept similarity (or semantic distance) to obtain related concepts and create new annotations. An ontology-mapping component creates new annotations based on existing mappings between different ontologies. For example, an annotation done with concept C0025202 (*melanoma*) in the NCI Thesaurus can be expanded to another one within SNOMED-CT because the UMLS metathesaurus provides the mapping information. The Annotator Web service is designed in manner that allows multiple semantic expansion components to be plugged-in, selected, and parameterized by a user when requesting the service. As the result of the second step, the direct annotations and several sets of semantically expanded annotations are extracted and returned to the user.

Annotations performed with the service have implicit semantics that declare that a given dataset (or record) is about (or references) a certain concept. Concepts are identified by UMLS Concept Unique Identifier (CUI) or National Center for Biomedical Ontology (NCBO) Uniform Resource Indicator (URI). The context of the annotation asserts whether the annotation is direct or semantically expanded. In the latter case, the component used to produce the expanded annotation is described along with the concept from which the new annotation is derived. For example, the annotation [C0431097-ISA_CLOSURE-C0025202] states that the given text was annotated with the concept C0431097 (*malignant melanocytic lesion*) using the is-a relations of the concept C0025202 (*melanoma*).

**Figure 3:**
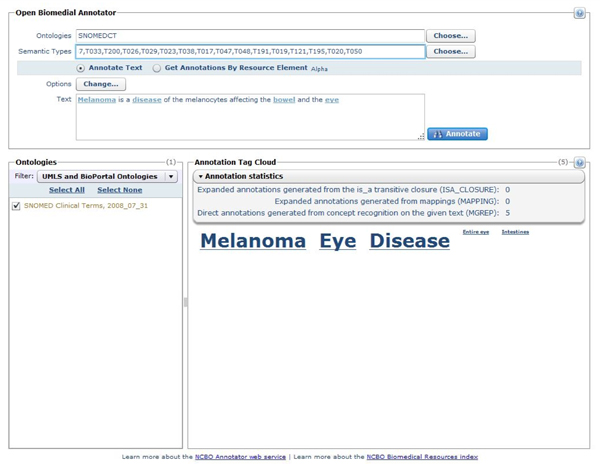
**User interface for accessing the Annotator Web service**. The figure shows a user interface for accessing the Annotator Web service. This UI enables users to figure out the best parameters to use in the programmatic service calls by allowing them to select different settings for ontologies to use, semantic types to restrict to as well as whether to use the semantic expansion components or not

Annotations can be returned to the user in different formats (text, tab delimited, or XML). The NCBO Annotator Web service is available and documented at [20]. The current implementation of the service uses a selection of 206 biomedical ontologies from UMLS and NCBO that gives a dictionary of 4021662 unique concepts and 7637125 terms. We also provide a rich user interface for users and developers to test different parameter setting before attempting to use the service programmatically. The Web-based user interface is shown in Figure 3.

### Evaluation of the service API

We have conducted tests simulating both single users and up to 10 concurrent users accessing the service API. For each test, we selected 400 records from Medline and submitted the title and abstract to the Annotator service. The records were selected at random. For each record, we measured the response time and recorded the number of words in the title and abstract. On average, the service responds in 1.8 seconds when the mean input word count is 180 words. The service responds in 2.3 seconds when the mean input word count is 280 words. When simulating 10 simultaneous users, the response time is between 4.5 and 5.0 seconds for 280 words.

## Discussion and future work

We identified the following considerations in selecting a concept recognizer for creating an automated ontology-based annotation service: (1) ability to work with non UMLS terminologies; (2) ability to work offline vs. online (annotation of user-submitted data as a service); (3) high speed as well as accuracy in terms of precision and recall.

By design, NIH's MetaMap is very tightly coupled with the UMLS. This makes mapping text to UMLS concepts very easy. However, generating a custom dictionary for annotation that uses concepts from outside UMLS is non-trivial. MetaMap requires the dictionary to be in a specific format with certain database tables always present. Some applications, such as the Open Biomedical Resources Index under development by the NCBO [1,17], use a number of different dictionaries from not only UMLS but also other sources for which terms are not present in the UMLS. Formatting such dictionaries into the format required by MetaMap is not always possible without a major effort. With respect to the input data, MetaMap is very adaptable and easy to customize. It does not require the input sources to be structured in any particular way.

In terms of speed of execution, MetaMap requires much more processing time than does Mgrep. For example, Mgrep can process 1/5^th ^of the data from ClinicalTrials.gov in 7 seconds, whereas MetaMap runs for over 8 minutes. This makes MetaMap unsuitable for developing an online annotation service. However the powerful lexical capability of MetaMap results in MetaMap finding about four times more concepts than Mgrep.

One of the standout features of Mgrep is its fast execution and scalability across all the dictionaries and data resources tested. However, Mgrep identifies a large number of concepts that are redundant – concepts recognized at the same position in the input string – and overall the number of unique concepts recognized is less than with MetaMap (Tables 3 and 4).

Mgrep is easily customizable to accept variation in the formats of both the input data and the dictionary, making it very easy to use for custom applications. It requires the dictionary to be in an easy to create two-column, tab-delimited file and similarly requires the resources to be in tab-delimited files. Mgrep places no rigid requirements on the structure and presence of concepts.

Mgrep shows higher precision than does MetaMap across most resources and dictionary types; possibly at the expense of some loss in recall. In the past we have used sampling along with a simplifying assumption – that at least one concept must be assigned to each record – to estimate recall [17]. In this study, that assumption is not always valid; e.g. There is no expectation that each record from Goldminer will be annotated with a biological process term. Hence we do not provide an estimate of recall. Recently a group from EBI has released a manually annotated corpus used in evaluating recall of recognizing disease names [21]. In future work, we can use this corpus for estimating recall of Mgrep for one dictionary – the 'diseases' dictionary.

Finally our use of a concept recognizer for building an annotator service distinguishes itself from previous efforts [5,22] for several reasons:

• The resulting service that can be integrated in current programs and workflows; current response times for the Annotator Web service are about 1.8 seconds for 180 words and 2.3 seconds for 280 words;

• Our service uses public ontologies both to create annotations and to expand them;

• Our service has access to one of the largest available sets of publicly available biomedical ontologies from the UMLS metathesaurus and the NCBO BioPortal repository. The current implementation of the service uses a selection of 206 biomedical ontologies that gives a dictionary of 4,021,662 unique concepts and 7,637,125 terms;

Future work will concentrate on three main areas that will determine the widespread adoption of the Annotator Web service: (1) enhancement of the concept-recognition step by using advanced natural languages processing techniques and eventually recognize 'relations,' (2) customizability of the service parameters, and (3) ability to plug-in concept recognizers other than Mgrep in the service. There are existing groups that already provide concept recognition as a service [22,23]. However, none of them have access to the scope of ontologies that our service has access to. We are actively working with several such groups to provide access to our ontologies for use in their concept recognition engines as well as to allow access to their concept recognizers within our Annotator Web service.

## Conclusion

MetaMap places a rigid constraint on the dictionary structure and cannot be used for applications that require dictionaries outside of the UMLS (such as those from the Open Biomedical Ontology library). Because of its slow speed, it cannot be used for many real-time applications or for applications in which either the data sources or the dictionary changes frequently, requiring recurrent reprocessing. Mgrep has extremely fast execution speed, but fewer concepts are recognized. If future versions of Mgrep provide the ability to generate lexical variants, recall would be enhanced and Mgrep could become the concept recognizer of choice for applications that need to process large datasets, that require large dictionaries, or that involve frequent reprocessing.

Ontology based annotation of biomedical data plays a crucial role for enabling data interoperability and the making of translational discoveries [1]. This situation is also true for e-science generally. The need to switch from the current Web to a semantic Web with semantically rich content annotated using ontologies has been clearly identified [24]. Meeting this need requires services (usable by humans and software agents) that can be integrated into existing data curation and annotation workflows.

We have used Mgrep to create a Web service for ontology based annotation of biomedical data. Our Annotator service has access to a large dictionary, which is composed of UMLS and NCBO ontologies. Our Annotator service is not limited to the syntactic recognition of terms, but also leverages the structure of the ontologies to expand annotations.

The annotator service workflow is currently used in a project within NCBO to annotate a large number of public biomedical resources [3]. The Annotator Web service is also available to the community for creating ontology-based annotation of their data. The service can be customized to their specific needs (in terms of annotation parameters and biomedical ontologies used).

## Competing interests

The authors declare that they have no competing interests.

## Authors' contributions

NHS conceived of the project, provided the scientific direction and wrote this manuscript, NB performed the evaluation of the concept recognizers. CJ built the Annotator Web service. DR and APC performed the evaluations. MAM contributed to the manuscript, provided critical feedback and supervision. All authors approved the final manuscript.
